# The association between prolonged capillary refill time and microcirculation changes in children with sepsis

**DOI:** 10.1186/s12887-024-04524-5

**Published:** 2024-01-20

**Authors:** Jaime Fernández-Sarmiento, Shirley Lamprea, Sofia Barrera, Lorena Acevedo, Catalina Duque, Manuela Trujillo, Valeria Aguirre, Carolina Jimenez

**Affiliations:** Department of Critical Care Medicine and Pediatrics, Universidad de La Sabana, Fundación Cardioinfantil-Institute of Cardiology, Bogotà, Colombia

**Keywords:** Septic shock, Endothelium, Fluid bolus, Resuscitation, Mortality

## Abstract

**Backgrounds:**

In children with sepsis, circulatory shock and multi-organ failure remain major contributors to mortality. Prolonged capillary refill time (PCRT) is a clinical tool associated with disease severity and tissue hypoperfusion. Microcirculation assessment with videomicroscopy represents a promising candidate for assessing and improving hemodynamic management strategies in children with sepsis. Particularly when there is loss of coherence between the macro and microcirculation (hemodynamic incoherence). We sought to evaluate the association between PCRT and microcirculation changes in sepsis.

**Methods:**

This was a prospective cohort study in children hospitalized with sepsis. Microcirculation was measured using sublingual video microscopy (capillary density and flow and perfused boundary region [PBR]—a parameter inversely proportional to vascular endothelial glycocalyx thickness), phalangeal tissue perfusion, and endothelial activation and glycocalyx injury biomarkers. The primary outcome was the association between PCRT and microcirculation changes.

**Results:**

A total of 132 children with sepsis were included, with a median age of two years (IQR 0.6–12.2). PCRT was associated with increased glycocalyx degradation (PBR 2.21 vs. 2.08 microns; aOR 2.65, 95% CI 1.09–6.34; *p* = 0.02) and fewer 4–6 micron capillaries recruited (*p* = 0.03), with no changes in the percentage of capillary blood volume (*p* = 0.13). Patients with hemodynamic incoherence had more PBR abnormalities (78.4% vs. 60.8%; aOR 2.58, 95% CI 1.06–6.29; *p* = 0.03) and the persistence of these abnormalities after six hours was associated with higher mortality (16.5% vs. 6.1%; *p* < 0.01). Children with an elevated arterio-venous CO_2_ difference (DCO_2_) had an abnormal PBR (aOR 1.13, 95% CI 1.01–1.26; *p* = 0.03) and a lower density of small capillaries (*p* < 0.05). Prolonged capillary refill time predicted an abnormal PBR (AUROC 0.81, 95% CI 0.64–0.98; *p* = 0.03) and relative percentage of blood in the capillaries (AUROC 0.82, 95% CI 0.58–1.00; *p* = 0.03) on admission. A normal CRT at 24 h predicted a shorter hospital stay (aOR 0.96, 95% CI 0.94–0.99; *p* < 0.05).

**Conclusions:**

We found an association between PCRT and microcirculation changes in children with sepsis. These patients had fewer small capillaries recruited and more endothelial glycocalyx degradation. This leads to nonperfused capillaries, affecting oxygen delivery to the tissues. These disorders were associated with hemodynamic incoherence and worse clinical outcomes when the CRT continued to be abnormal 24 h after admission.

**Supplementary Information:**

The online version contains supplementary material available at 10.1186/s12887-024-04524-5.

## Introduction

Sepsis continues to be a significant public health problem worldwide [1]. High mortality rates have been reported in children, especially in middle and low-income countries [2]. An explanation for these unsatisfactory outcomes is often the rapid and severe circulatory involvement in patients with sepsis. Early recognition and appropriate fluid resuscitation could improve the outcomes. However, due to its broad spectrum of clinical presentation, the signs of hypoperfusion are often not recognized. Some biomarkers, like lactate, are not always available and tend to be a late indicator of tissue hypoxia [3]. Capillary refill time (CRT) is a useful, easily accessible and universally available marker of peripheral perfusion, which worsens during circulatory failure in critically ill patients [4].

In sepsis, CRT is an indicator of disease severity and could be used to monitor treatment response [5]. However, this clinical parameter can be affected by a few circumstances, including room temperature, age, technique or the extremity in which it is measured [6]. Abnormal skin perfusion is assumed to indicate high adrenergic tone and low blood flow in the microcirculation. The capillary blood volume generally depends on what is known as the capillary “driving pressure” [7, 8]. This concept refers to the difference between the precapillary arteriole/precapillary sphincter pressure and the venous pressure. The capillaries lack innervation and have no smooth muscle layer. The muscle layer is described as an intermittent smooth muscle layer up to the meta-arterioles (10–20 microns). This means that the amount of flow in the capillary is determined by other factors that can be altered in sepsis [8]. Due to these anatomical characteristics, a capillary can be irrigated by multiple arterioles. Under disease conditions, this is an important physiological mechanism because capillary blood flow can increase by 200–500% without any significant change in systemic blood pressure [9, 10].

In children with sepsis, CRT abnormalities depend on a fine balance between vasoconstricting and vasodilating substances as well as capillary permeability [11, 12]. We do not know what changes occur in children’s microcirculation, endothelium and glycocalyx in patients with prolonged CRT. With the technology available today, we can deepen our knowledge of this clinical tool and have a more detailed understanding of the microcirculation changes in terms of flow, capillary blood vessel density or glycocalyx abnormalities in critically ill patients. Understanding these microcirculation changes associated with prolonged capillary refill will allow this tool to be used at the patients’ bedside to guide treatment, as has recently been described in adults with sepsis [5]. Furthermore, it will help explain the changes in capillary flow and density associated with dissociation between macro and microcirculatory variables (a phenomenon known as hemodynamic incoherence) and the possible related outcomes in children with sepsis [5, 11, 12]. The primary objective of this study was to evaluate the association between prolonged CRT (PCRT) in children with sepsis and microcirculatory changes as determined by sublingual video microscopy and plasma biomarkers. The secondary objective of this study was to determine associations between PCRT and clinical outcomes in children with sepsis.

## Materials and methods

### Ethical approval

The Safety, Quality, Management and Research Committee at Fundación Cardioinfantil approved this study (IRB-DDI-352–2019). This study was also approved by the Department of Pediatrics and Critical Care at Universidad de La Sabana in Bogotá, Colombia (MED-256–2019). Written informed consent was obtained from the parents or legal guardians of all enrolled children. Procedures were followed in accordance with the ethical standards of the Fundación Cardioinfantil-IC IRB and with the Helsinki Declaration of 1975.

### Study design and context

This was a prospective, observational study in children hospitalized for sepsis who required admission to the pediatric intensive care unit (PICU) at Fundación Cardio-Infantil in Bogotá, Colombia, between January 2021 and December 2022. Children one month to 18 years old with sepsis or septic shock who were transferred to the PICU due to hemodynamic or respiratory instability were included. In addition, simultaneous sublingual video microscopy and endothelial activation, glycolcalyx degradation and inflammation biomarker measurements were performed within the first 6 h after admission and 24 h following admission. These data were recorded at the same time as video microscopy, on admission to the study and 24 h later. Patients with hyperglycemia, diabetic ketoacidosis, head trauma, continuous renal support therapy and children with congenital heart disease were excluded.

### Definition of variables

Sepsis was defined as a syndrome characterized by potentially fatal organ dysfunction caused by a dysregulated host response to infection. Septic shock was defined as sepsis with particularly severe circulatory, cellular and metabolic abnormalities, according to the recently recommended definitions [2]. Standardized measurement of CRT was performed by applying firm pressure on the anterior surface of the distal phalange of the right index finger using a glass slide with the hand at heart level [5]. Pressure was increased until the skin was white. This pressure was maintained for 10 s, then a specific timer for this study was activated and the time taken to return to a pink color (considered normal) was recorded for each patient. A CRT longer than two seconds was considered prolonged [4, 11]. Acute kidney injury was defined as an abnormal creatinine for the patient’s height, according to the Schwartz equation [13]. Hemodynamic incoherence was defined as patients with an arterial pressure and heart rate within normal limits for their age but with a capillary refill of more than two seconds [12, 14, 15]. Serum lactate was considered abnormal if greater than 2 mmol/L [2, 9]. Arterial and venous gases were measured at the same time as the microcirculation, with a venous-arterial CO_2_ difference greater than 6 mmHg considered abnormal [14]. Metabolic acidosis was defined as a serum bicarbonate level less than 15 mEq/L.

### Microcirculation, endothelial activation and glycocalyx degradation measurement

The definition of microcirculation used was developed according to the consensus on sublingual microcirculation, based on the information provided by video microscopy [15]. Capillaries were defined as blood vessels fed by several arterioles which were less than 10 microns in diameter, with single file red blood cells. Meta-arterioles were defined as vessels with a 10 to 20 microns diameter. Microcirculation and endothelial glycocalyx degradation were evaluated in vivo using video microscopy (*Glycocheck System ®—Microvascular Health Solutions Inc 2014, Salt Lake City, UT, USA*), and this was conducted at the same time as CRT measurement. This device measures the sublingual microcirculation, evaluating 4–25 micron diameter vessels using a dark field camera (*CapiScope, HVCS, KK* Technology United Kingdom) which emits stroboscopic green light diodes that detect red blood cells by reflection. This is a high resolution (720 pixel) camera, which is also able to amplify the images slightly more than 300 times. In addition, the machine has software (*Glycocheck System®*) that analyzes the measurements from high-quality images. In order to do this, it defines 10-micron vascular segments and records 40 frames (300 green segments, which are the ones with complete measurement). With small movements, approximately 3,000 vascular segments can be recorded in each imaging session. Once the images are taken, the machine’s software analyzes and processes them and provides information regarding microcirculation, vascular density, blood flow and the endothelial glycocalyx. The video microscope measures the distance between the red blood cells and the endothelial glycocalyx, which has been called the “perfused boundary region” (PBR, in microns). This variable is inversely related to the endothelial glycocalyx dimensions. In healthy individuals, including children, the normal PBR is considered to be less than 2.0 microns [16, 17]. As previously mentioned, the machine also reports capillary blood volume variables, the percentage of capillary recruitment blood volume (PPV -*proportion of perfused blood vessels over the total number of vessels*) and the capillary density of 4–6-micron vessels (CD 4-6 m) as well as how it compares with 7–20 micron blood vessels. The CD-4-6 s indicate the capillary network’s ability to carry nutrients to the organs and tissue cells. A higher number suggests a better capacity for direct delivery of nutrients to the tissues. Video microscopes have proven to have low inter and intra-observer variability, with high concordance in different clinical settings (intraclass correlation coefficient of 0.77; 95% CI 0.52–0.87) [18]. We evaluated plasma angiopoietin-2 levels as a biomarker of vascular endothelial cell activation (Ang-2 / Human Angiopoietin 2 ELISA Kit ANG 2; ab99971, Abcam Lab). Plasma syndecan-1 concentration was measured as the biomarker for endothelial glycocalyx degradation (value less than 80 mg/dL was considered normal / Human Syndecan-1 ELISA Kit CD138; ab46506; Abcam Lab) [19], along with plasma endocan concentrations (Human Endocan ELISA Kit Human ESM1; ab213776; Abcam Lab) [19, 20]. The samples were 100 µl of citrated plasma which were stored at (-) 20 °C and processing using the enzyme-linked immunosorbent assay (ELISA) method.

### Variable categories

Demographic information retrieved included age, weight, sex and date of admission. Clinical data included vital signs, fluid balance, the need for mechanical ventilation and vasoactive drug infusions. The clinical outcome variables evaluated included organ failure, using the Pediatric Logistic Organ Dysfunction-2 (PELOD-2) score, which was calculated within six hours of admission to the PICU. In addition, disease severity was assessed using the Pediatric Index of Mortality-2 (PIM-2) on PICU admission, and mortality was evaluated at PICU discharge as a dichotomous variable (living or deceased). Other outcome variables included the venous-arterial CO_2_ difference, serum lactate level and PICU stay. The biomarker evaluated for endothelial activation was Ang-2, with syndecan-1, endocan and the PBR taken with video microscopy used to evaluate glycocalyx degradation, all taken on PICU admission. Lab tests included electrolytes, blood urea nitrogen, creatinine, serum lactate, serum bicarbonate, serum albumin, D-dimer and biomarkers of inflammation (C-reactive protein, procalcitonin, ferritin). Arterial blood was obtained from an arterial line and venous blood was drawn from a central venous catheter located in the subclavian or internal jugular vein, at the cavoatrial junction, and these measurements were performed at all study times (Rapidlab 1265, (15,630 series)/Siemens 2010 ® gas analyzer).

### Outcomes

The primary outcome was the change in microcirculation (density, flow and endothelial glycocalyx disruption) between groups with and without prolonged capillary refill time. The secondary outcomes were abnormalities in the blood venous-arterial CO_2_ difference, serum lactate levels, and clinical outcomes of interest between the groups.

### Statistical methods

Univariate analysis was performed, reporting each cohort with and without CRT abnormalities. Categorical variables were reported as proportions and numerical variables were reported as means or medians, according to their distribution, with standard deviation or interquartile range, respectively. In the bivariate analysis, Pearson’s Chi^2^ or Fisher’s exact test were used for categorical variables. Student’s t-test or Wilcoxon’s test were used for numerical variables, depending on their distribution. Given the methodological design, we attempted to control for confounding covariables from the design stage (excluding factors reported to injure the glycocalyx) [10, 16, 17]. A multivariate analysis was run, adjusting the variables for the disease severity (PIM-2 scale), age and need for vasoactive drugs, which could be confounding variables. Binary logistic regression was done, including variables with biological plausibility (fluid overload, PBR, organ failure score – PELOD-2) and those which met the Hosmer–Lemeshow criteria on bivariate analysis. The model was constructed using the forward method and was adjusted with the omnibus test. A receiver operating characteristic (ROC) curve was used to evaluate the predictive capacity of PCRT for microcirculation disorders and glycocalyx degradation evaluated by PBR. The aim was to detect an ROC-AUC of 0.8 with a power of 0.8 and an α-risk of 0.05. Two-sided analyses were performed with a p value less than or equal to 0.05 considered to be statistically significant. Analyses were performed using the R V.3.3.3 (www.r-project.org) statistical package.

## Results

A total of 132 patients with sepsis or septic shock were included during the study period (Table 1). The median age was two years (IQR 0.6–12.2) for the two groups. The participants were distributed similarly by sex (63/132; 47% females). Of these, 43/132 (32.5%) had prolonged capillary refill. The main cause of PICU admission was respiratory problems. Of the patients with septic shock, 55.8% (24/43) had prolonged capillary refill and 28% (12/43) had hemodynamic incoherence on admission (Supplementary material).

**Table 1:** Population characteristics according to the capillary refill time on admission

**Characteristic**	**Total** ***n* = 132**	**Capillary refill less than 2 sec** *n*** = 89**	**Capillary refill greater than** **2 sec** *n*** = 43**	***P* value**
**Age, years (IQR)**	2.0 (0.6–12.2)	1.9 (0.6–13.0)	4.0 (0.5–11.0)	0.83^a^
**Weight, kg (IQR)**	10.9 (8.7–30.0)	10.5 (6.9–35.2)	14.0 (6.4–27.8)	0.69^a^
**Female Sex (%)**	63.0 (47.7)	40.0 (44.9)	23.0 (53.5)	0.35^b^
**Days in PICU (IQR)**	11.0 (6.5–19.0)	11.0 (6.5–18.0)	11.0 (6.5–26.0)	0.69^a^
**Focus of Infection (%)** Respiratory Gastrointestinal Genitourinary CNS Other	58.0 (44.6%) 44.0 (33.4%) 2.0 (1.5%) 7.0 (5.3%) 21.0 (15.9%)	42.0 (47.2%) 30.0 (33.7%) 1.0 (1.1%) 4.0 (4.5%) 12.0 (13.4%)	16.0 (37.2%) 14.0 (32.5%) 1.0 (3.1%) 3.0 (7.0%) 9.0 (20.1%)	0.65^b^
**Sepsis classification (%)** Severe sepsis Septic shock	54.0 (41.2%) 78.0 (58.8%)	36.0 (40.4%) 53.0 (59.6%)	18.0 (41.9%) 25.0 (58.1%)	0.79^b^
**PRISM III (IQR)**	15.0 (9.0- 21.0)	16.0 (9.0–20.0)	15.0 (10.0–23.0)	0.91^a^
**PIM-2 (IQR)**	18.1 (8.9–31.6)	16.6 (7.0–29.2)	21.7 (14.2- 35.5)	0.04^a^
**PELOD-2 Score (IQR)**	8.0 (3.0–10.0)	7.0 (3.0- 9.0)	9.0 (5.0 -11.0)	0.07^a^
**Lactate mmol/L (IQR)**	1.2 (0.8 to 1.8)	1.2 (0.8 -1.8)	1.2 (0.9 -1.7)	0.45^a^
**Glucose (SD) mg/dL**	109.2 (91.9 -137.4)	114.0 (91.5–141.6)	108.8 (92.3–131.9)	0.93^a^
**DCO**_**2**_** mmHg (IQR)**	5.1 (2.8–7.1)	5.1 (2.8–7.1)	5.1 (2.9–7.5)	0.73^a^
**Ferritin mg/dL (IQR)**	431.1 (180.3–1698.5)	427.1 (189.0–1261.8)	460.2 (148.8–2314.5)	0.88^a^
**CRP mg/dL (IQR)**	5.1 (2.0–9.6)	4.2 (1.6–8.6)	5.8 (2.6–17.3)	0.02^a^
**D-dimer mg/L (IQR)**	3.2 (1.5–6.2)	2.9 (1.2–4.0)	7.8 (2.9–9.7)	0.09^a^
**Procalcitonin g/dL (IQR)**	1.2 (0.4–5.2)	1.0 (0.4–4.2)	2.4 (0.4–7.6)	0.11^a^
**Creatinine mg/dL (IQR)**	0.4 (0.4–0.6)	0.4 (0.4–0.6)	0.4 (0.4–0.6)	0.77^a^
**Vasoactive Score**	12.3 (4.5–28.4)	11.5 (4.0–24.1)	12.3 (5.9–29.7)	0.30^a^
**Mechanical Ventilation (%)**	76 (57.6%)	48 (53.9%)	28 (65.5%)	0.23^b^
**Mortality (%)**	14.0 (10.6%)	8.0 (9.0%)	6.0 (14.0%)	0.38^b^

*PIM-2* Pediatric Index of Mortality-2, *PELOD* Pediatric Logistic Organ Dysfunction-2, *DC0*_*2*_ venous-arterial C0_2_ difference, *CRP* C-reactive protein

^a^ Mann–Whitney U test for quantitative variables with a non-normal distribution

^b^ Chi^2^ test for categorical variables or Fisher's exact test

### Primary outcome

**Fig. 1:**
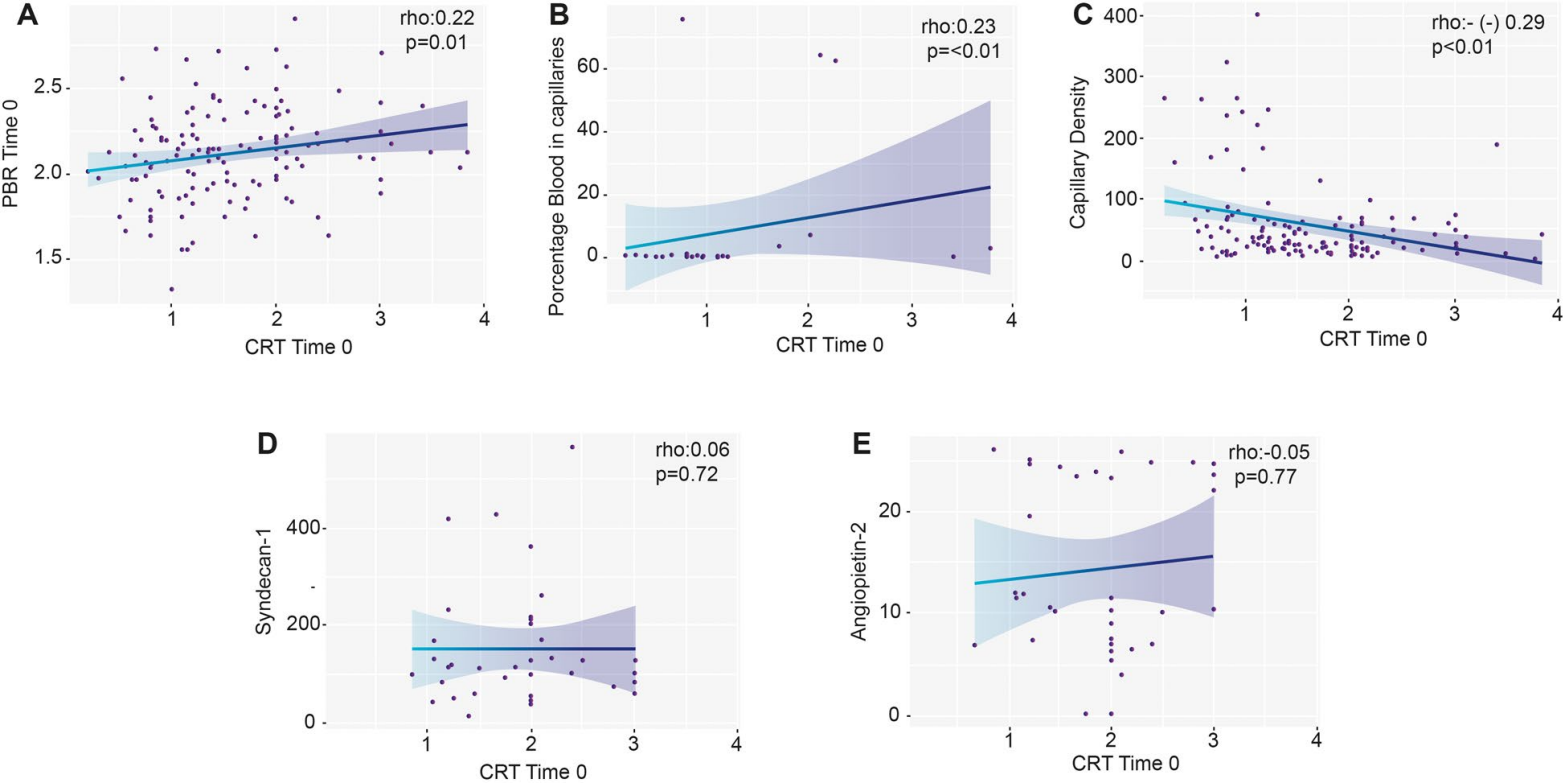
Correlation between capillary refill time and microcirculatory findings by videomicroscopy and biomarkers. CRT capillary refill time; PBR: perfunded boundary region. Time 0 is on admission to PICU. rho Spearman's correlation coefficient. The shaded line corresponds to the confidence intervals

**Fig. 2:**
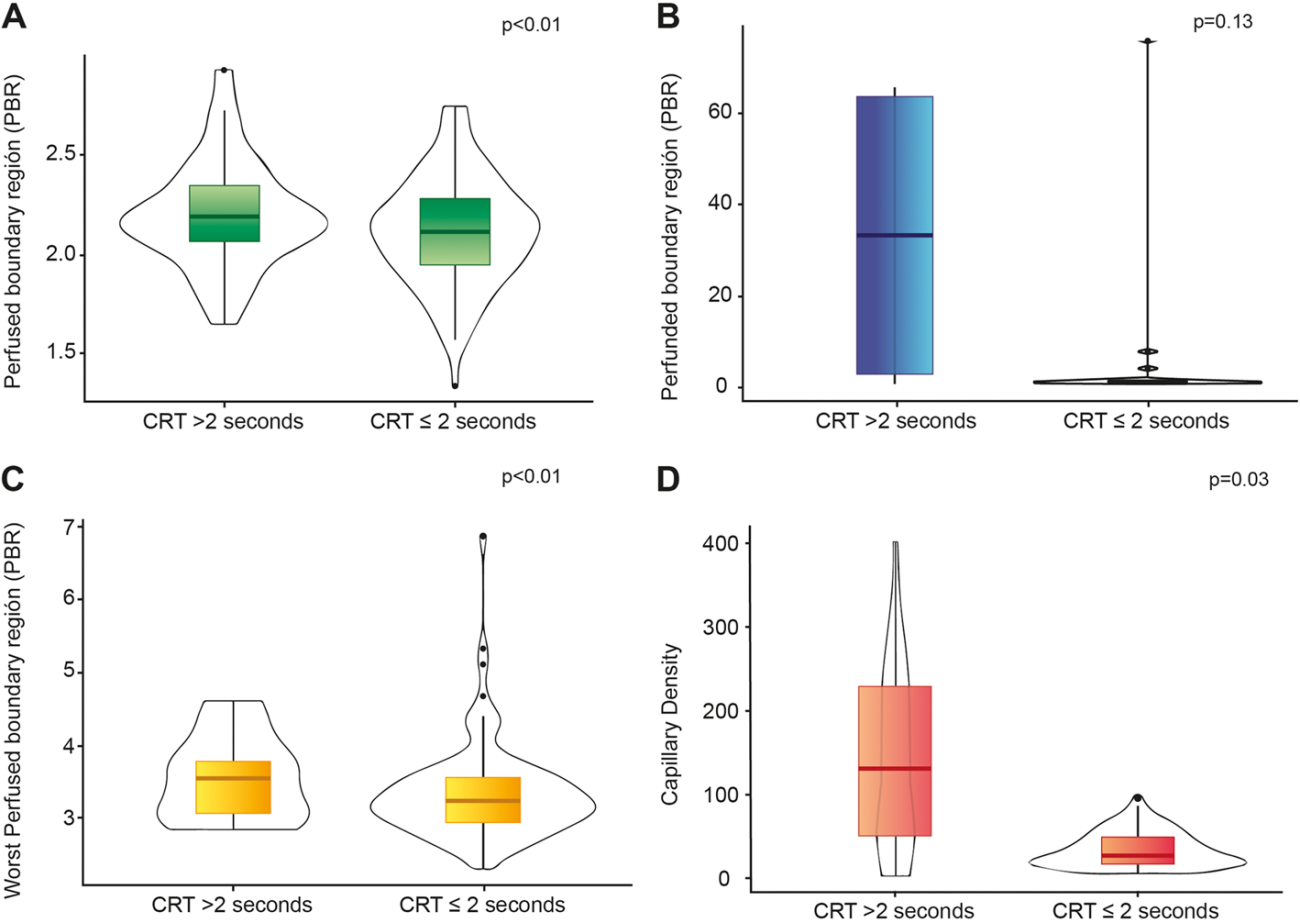
Changes in microcirculation associated with prolonged capillary refill time at the time of admission. CRT capillary refill time; PBR:perfunded boundary region. U-Mann–Whitney test for figures **A** to **D**. Box plots show the medians and interquartile range

We found a weak correlation between PCRT and PBR (rho 0.22; *p* = 0.01) as well as the percentage of blood volume in each capillary (rho 0.23; *p* = 0.01) (Fig. 1). Likewise, there was an inverse correlation between PCRT and the number of small capillaries (4–6 microns) recruited (rho (-) 0.30; *p* < 0.01). A capillary refill greater than two seconds was found in 81.4% (35/43) of the patients with an abnormal PBR on admission (aOR 2.65, 95% CI 1.09–6.34; *p* = 0.02). Patients with PCRT had a lower 4–6 micron capillary density on admission (Table 2). However, we found no differences in the percentage of capillary blood volume (aOR 1.1, 95% CI 0.98–1.02; *p* = 0.97) after adjusting for disease severity. Patients with normal blood pressure but prolonged capillary refill were more likely to have an abnormal PBR on admission (78.4% vs. 60.8%; aOR 2.58, 95% CI 1.06–6.29; *p* = 0.03) (Fig. 2). Six hours after admission, the patients who still had hemodynamic incoherence had a higher mortality (16.5% vs. 6.1%; *p* < 0.01) than those who did not.

**Table 2:** Microcirculation changes and prolonged capillary refill time on admission to the PICU

Microcirculation Variables	Total *N* = 132	Capillary refill less than 2 sec *N* = 89	Capillary refill greater than 2 sec *N* = 43	*P* value
PBR (microns) (SD)	2.12 (0.27)	2.08 (0.27)	2.21 (0.26)	0.01^a^
PBR Flow Corrected (microns) (SD)	2.11 (0.51)	2.10 (0.49)	2.01 (0.55)	0.93^a^
Worst PBR microns (IQR)	3.26 (2.99 -3.61)	3.23 (2.97 -3.54)	3.28 (2.99–3.63)	0.61^b^
Capillary Density 4–6 microns (IQR)	36.8 (18.9–64.9)	39.6 (21.0–69.9)	29.5 (15.8–49.7)	0.03^b^
Capillary Blood Volume (%) (IQR)	63.2 (19.0–83.3)	62.4 (10.3–83.2)	66.5 (36.1–85.5)	0.13^b^
Syndecan-1 ng/mL (IQR)	116.8 (82.8–180.8)	115.2 (80.7–142.0)	129.7 (82.8–207.8)	0.55^b^
Angiopoietin-2 ng/mL (IQR)	11.6 (7.3–23.8)	12.1 (10.5–24.1)	10.3 (7.0–23.4)	0.20^b^
Endocan ng/mL (SD)	2.4 (1.6)	2.5 (1.9)	2.2 (1.4)	0.64^a^

*PBR* perfused boundary region

^a^ Student’s t-test

^b^ Mann–Whitney U test

Twenty-four hours after admission, the patients with prolonged capillary refill had fewer 4–6 micron capillaries recruited (*p* = 0.03) despite having a more than 10% positive fluid balance more often (36.1% [13/36] vs. 11.7% [11/94]: aOR 4.26, 95% CI 1.68–10.76; *p* < 0.01). However, there was no difference in the percentage of blood volume reaching all the capillaries (*p* = 0.13). We were unable to show an association between PCRT and syndecan-1 (*p* = 0.22), Ang-2 (*p* = 0.19), or endocan (*p* = 0.87) abnormalities, 24 h after admission. Patients with septic shock and prolonged capillary refill were more likely to have endothelial glycocalyx degradation evaluated with PBR (40.7% [22/54] vs. 9.1% [2/22]; aOR 2.56, 95% CI 1.05–6.19; *p* = 0.03) than patients without shock and prolonged capillary refill.

### Secondary outcomes

No correlation was found between CRT on admission and DCO_2_ (rho 0.03; *p* = 0.74). However, there was a correlation between these variables at 24 h (rho 0.2; *p* = 0.03). Altogether, 76.1% (35/46) of the patients with elevated DCO_2_ had an abnormal PBR (aOR 1.13, 95% CI 1.01–1.26; *p* = 0.03), regardless of age or PIM-2 severity score on admission. These changes persisted after 24 h. Patients with a DCO_2_ greater than 6 also had a lower density of small capillaries (*p* < 0.05) recruited on admission. We did not find an association between elevated DCO_2_ and serum lactate (*p* = 0.99) or metabolic acidosis (*p* = 0.06). We found a correlation between PCRT on admission and the lactate level (rho 0.25; *p* < 0.01) but not after 24 h (rho 0.09; *p* = 0.37). A normal CRT was associated with a lower PELOD-2 (aOR 0.88, 95% CI 0.81–0.98; *p* = 0.02) on admission, and after 24 h it was a good predictor of a shorter hospital stay (aOR 0.96, 95% CI 0.94–0.99; *p* < 0.05) regardless of age and PIM-2.

**Fig. 3:**
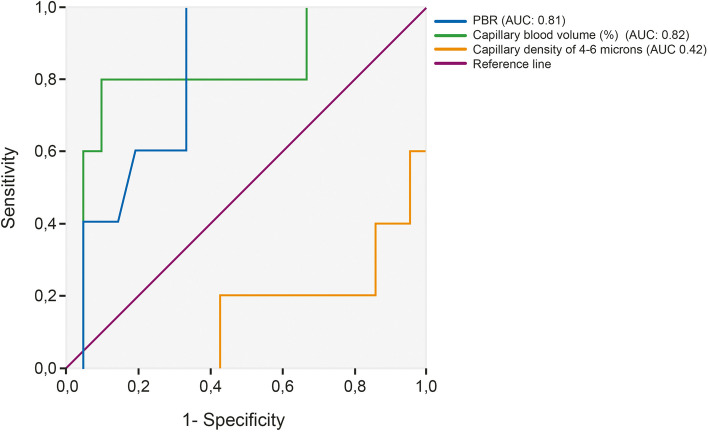
Receiver operating curve (ROC) of prolonged capillary refill and changes in microcirculation on admission. PBR: perfused boundary region. AUC: area under the curve

We found that a CRT greater than two seconds on admission was a good predictor of an abnormal PBR (AUROC 0.81, 95% CI 0.64–0.98; *p* = 0.03) and the relative percentage of blood in the capillaries (AUROC 0.82, 95% CI 0.58–1.00; *p* = 0.03 (Fig. 3). Children with hemodynamic incoherence had prolonged capillary refill 24 h after admission despite fluid balances greater than 10% (*p* < 0.01). We found no association between PCRT and metabolic acidosis (*p* = 0.67), the need for vasoactives (*p* = 0.27), duration of mechanical ventilation (*p* = 0.58) or mortality (*p* = 0.60).

## Discussion

In this study, we found that children with sepsis and PCRT had profound abnormalities in their microcirculation, endothelium and glycocalyx. Our main findings are that *1*) children with PCRT had fewer CD-4-6 m vessels, indicating less capacity to deliver oxygen and nutrients to the tissues; *2*) while we found no changes in the compensatory percentage of capillary blood flow, we did find a higher likelihood of microcirculatory structural abnormalities like endothelial glycocalyx damage; *3*) the persistence of these microcirculatory abnormalities (PCRT) when macrocirculatory variables normalized (a phenomenon known as hemodynamic incoherence) was associated with greater mortality; *4*) a normal capillary refill time on admission was associated with less multiple organ failure and, at 24 h, with a shorter hospital stay.

Capillary refill changes are associated with microcirculation dysfunction [21, 22]. In humans, DeBacker et al. [23] showed that sublingual microcirculation was abnormal in patients with sepsis. Many subsequent studies have replicated these findings in patients of all ages, including children [24, 25]. These studies have found that the density of perfused capillary vessels is lower in sepsis, and perfusion heterogeneity increases. This leads to nonperfused capillaries, affecting oxygen delivery to the tissues. In our study, we found that these microcirculation changes measured with sublingual video microscopy were associated with PCRT in children with sepsis.

We found that patients with PCRT had a higher risk of endothelial glycocalyx degradation. This layer of glycosaminoglycans, proteoglycans and glycoproteins on the endothelial surface has been reported to be abnormal in patients with sepsis. Glycocalyx degradation contributes to (micro) vascular dysfunction, favoring the adhesion of circulating cells, microthrombosis and increased permeability [26, 27]. The severity of glycocalyx injury has been associated with a poor clinical outcome in patients with sepsis [28]. We also found that the risk of glycocalyx injury and degradation in patients with PCRT was greater in children in shock with persistent hemodynamic incoherence after the initial PICU interventions.

In this regard, hemodynamic incoherence is an increasingly recognized clinical condition in patients with sepsis. When there are hemodynamic abnormalities, fluid resuscitation improves the macrocirculation but not necessarily microcirculation parameters [29]. Hemodynamic incoherence has been associated with late changes in microcirculation and worse outcomes in patients with sepsis [30, 31]. Four types of microcirculation abnormalities have been described when coherence is lost, all related to poor tissue oxygen extraction capacity [15]. In our study, we found that patients with prolonged capillary refill had heterogeneity in capillary density and less recruitment of small capillaries. These are characteristics of type 1 microcirculatory disorders (characterized by capillary density heterogeneity) and loss of hemodynamic coherence, which has been described in patients with sepsis [15]. Persistent hemodynamic incoherence after six hours was associated with microcirculation changes and PCRT. This hemodynamic incoherence can be explained by a reduced microcirculatory driving pressure (due to an elevation of capillary venous pressure), which could explain how fluid overload is associated with worse outcomes in critically ill children. These findings may be very useful, especially in middle and low-income settings. Identifying children with persistent PCRT and possible loss of hemodynamic coherence after fluid resuscitation could help rationalize fluid resuscitation, identify fluid therapy response phenotypes, and potentially improve outcomes.

It has been found that resuscitation based on the administration of fluid boluses, blood products or vasoactives could optimize oxygen delivery and the recovery of hemodynamic coherence [30]. However, selecting fluid therapy for children guided by microcirculation goals has not yet been studied. A recent study in adults with septic shock found that fluid boluses could be safely discontinued in patients with no response to tissue perfusion indicators [32]. Castro et al. [33] evaluated the impact of a fluid resuscitation strategy guided by CRT, simultaneously measuring sublingual microcirculation. They found improved regional flow parameters, and fluid resuscitation goals were reached faster. Pranskunas et al. [34] found that fluid boluses may only be useful when the microcirculation flow is low, with no benefits from fluid therapy if the microcirculation flow is normal. These authors, using organ hypoperfusion substitutes (for example, lactate, tachycardia, hypotension, oliguria), found that abnormalities in these variables could have normal or reduced microcirculatory flow. However, only patients with diminished microcirculatory flow benefited from fluid administration [35, 36]. CRT could be a clinical tool to guide fluid resuscitation in children with sepsis and identify different patient phenotypes with a better or worse response to fluid therapy, especially if we consider our findings of the association between PCRT and microcirculation abnormalities. Prospective studies in children with sepsis are needed to evaluate CRT-guided fluid therapy, understanding its importance as an indicator of abnormal tissue perfusion and microcirculation.

We believe our study has several limitations. First, we consider that the data presented here represent the experience of a single referral center which admits highly complex pediatric patients. This could bias the data obtained, as the patients were sicker and we did not include a control group that could have enriched the analyses. Also, the type of design used in our study could have limitations in analyzing physiological variables. Our results should be considered hypothesis generators on how PCRT could be useful in identifying the phenotypes of patients with greater or lesser microcirculation involvement. Another limitation of our study is that capillary refill can have high inter-observer variability. We sought to reduce this with a standard technique using a glass slide, which must be validated in children, as it was extrapolated from studies in adults [31].

## Conclusions

In this study, we found a significant association between PCRT and changes in the microcirculation of children with sepsis. Specifically, these patients have fewer small capillaries recruited, increased redistribution of the blood flow toward these capillaries and a higher risk of endothelial glycocalyx degradation. These abnormalities were associated with hemodynamic incoherence and worse clinical outcomes when the abnormal CRT persisted 24 h after admission to intensive care. The CRT may be a good surrogate of microcirculation abnormalities in children and may be a simple and accessible tool for monitoring and evaluating interventions in patients with sepsis.

### Supplementary Information

**Additional file 1.** Microcirculation changes on admission to the PICU in patients with and without hemodynamic incoherence.

## Data Availability

The data that support the findings of this study are available from corresponding author (Jaime Fernández-Sarmiento) but restrictions apply to the availability of these data, which were used under license for the current study, and so are not publicly available. Data are however available from the authors upon reasonable request and with permission of Jaime Fernández-Sarmiento.

## Abbreviations

Ang-2: Angiopoietin-2
CI: Confidence interval
CD 4-6 m: Capillary density of 4–6 micron vessels
DCO_2_: Venous-arterial CO_2_ difference
ICU: Intensive care unit
IQR: Interquartile range
PBR: Perfunded Boundary Region
PCRT: Prolonged Capillary Refill Time
PPV: Proportion of Perfused Blood Vessels
ROC: Receiver Operating characteristic Curve
AUC: Area Under the Curve
