# Supplementary material for: The association between prolonged capillary refill time and microcirculation changes in children with sepsis
Source: BMC Pediatr. 2024 Jan 20;24:68. doi: 10.1186/s12887-024-04524-5 (PMC10799439; doi:10.1186/s12887-024-04524-5)
Supplement: Supplementary file 1 — Additional file 1. Microcirculation changes on admission to the PICU in patients with and without hemodynamic incoherence. [file 12887_2024_4524_MOESM1_ESM.docx]

**Supplementary material.**

**Microcirculation changes on admission to the PICU in patients with and without hemodynamic incoherence**

| Microcirculation Variables | | Total  N=132 | Hemodynamic Incoherence*  N=12 | Hemodynamic Coherence  N=120 | P value |
| --- | --- | --- | --- | --- | --- |
| PBR (microns) (SD) | 2.12 (0.27) | | 2.3 (0.2) | 2.1 (0.3) | 0.04^a^ |
| PBR Flow Corrected (microns) (SD) | 2.11 (0.51) | | 2.4 (0.6) | 2.1 (0.5) | 0.03^a^ |
| Worst PBR microns (IQR) | 3.3  (3.0 -3.6) | | 3.4  (3.1-3.7) | 3.2  (3.0-3.6) | 0.26 ^b^ |
| Capillary Density 4-6 microns (IQR) | 36.8  (18.9-64.9) | | 29.9  (14.8-54.5) | 41.3  (20.8-67.6) | 0.04 ^b^ |
| Capillary Blood Volume (%) (IQR) | 63.2  (19.0-83.3) | | 57.4  (35.7-81.2) | 69.1  (13.6-83.8) | 0.801^b^ |
| Syndecan-1 ng/mL (IQR) | 116.8  (82.8-180.8) | | 129.7  (98-215.8) | 107.5  (72.9-133.4) | 0.308^b^ |
| Angiopoietin-2 ng/mL (IQR) | 11.6  (7.3-23.8) | | 10.2  (7.1-20.9) | 17.8  (10.6-24.5) | 0.08^b^ |
| Endocan ng/mL (SD) | 2.4 (1.6) | | 2.4 (1.7) | 2.4 (1.6) | 0.99^b^ |

*PBR:* perfused boundary region.  ^a^ T-student test ^b^ U-Mann Whitney *Total patients included with hemodynamic incoherence (blood pressure and heart rate normal for their age with capillary refill greater than 2 seconds). At the time of admission, 28% (12/43) had hemodynamic incoherence.
